# Shared decision making for patients needing dentofacial orthopedics, orthognathic surgery, and conventional non-surgical fixed appliance therapy: a comparison between Pakistani patients’ and clinicians’ perspective

**DOI:** 10.1590/2177-6709.29.4.e242443.oar

**Published:** 2024-09-02

**Authors:** Fatima Naz NAJAM, Waqar JEELANI, Maheen AHMED, Mirza Ezaaf SHUJA

**Affiliations:** 1 Bakhtawar Amin Medical and Dental College, Dental School, Department of Orthodontics (Multan, Pakistan).

**Keywords:** Shared decision making, Dyadic OPTION scale, Patient involvement, Tomada de decisão compartilhada, Escala diádica OPTION, Envolvimento do paciente

## Abstract

**Introduction:**

Shared decision making (SDM) involves presenting patients with relevant information about a health issue and reaching a clinical decision based on their preferences. However, its application in orthodontics lacks documentation.

**Objective:**

This study aimed to assess and compare the perspectives of patients and clinicians on SDM in different cases.

**Methods:**

A cross-sectional study was conducted at a tertiary care hospital in Pakistan, involving 90 patients categorized into three groups (dentofacial orthopedics, orthognathic surgery, and conventional non-surgical fixed appliance treatment). Following clinical assessment and treatment plan discussions, patients and clinicians completed a 12-item dyadic observing patient involvement in decision making (OPTION) questionnaire, to gauge their perspectives on SDM. Mean OPTION scale scores were compared using paired sample *t*-tests between clinicians and patients, and intergroup comparisons utilized paired sample *t*-tests and Pearson correlation coefficients.

**Results:**

OPTION scores were similar between patients/parents and clinicians. However, statistically significant differences were found regarding the questions about “different sources of information”, “different options (including the possibility of doing nothing)” and “concerns regarding management”, with the patients giving overall lower OPTION scores. Patients gave lower SDM OPTION scores for conventional orthodontic treatment, but higher scores for orthopedic and orthognathic surgery, as compared to the clinicians.

**Conclusions:**

The current study revealed an overall consensus in the mean total scores of OPTION scales between patients and clinicians. However, when stratified, patients showed higher SDM scores for orthopedic and orthognathic cases, and lower scores for conventional orthodontic treatment.

## RESUMO

**Introdução:**

A tomada de decisão compartilhada (TDC) envolve apresentar aos pacientes informações relevantes sobre um problema de saúde e chegar a uma decisão clínica com base em suas preferências. Porém, seu uso em Ortodontia carece de literatura.

**Objetivo:**

Este estudo teve como objetivo avaliar e comparar as perspectivas de pacientes e dentistas paquistaneses sobre a TDC em diferentes casos.

**Métodos:**

Um estudo transversal foi realizado em um hospital terciário no Paquistão, envolvendo 90 pacientes categorizados em três grupos (ortopedia dentofacial, cirurgia ortognática, e tratamento convencional não cirúrgico com aparelhos fixos). Após a avaliação clínica e as discussões sobre o plano de tratamento, os pacientes e os dentistas preencheram um questionário com 12 itens, de observação do envolvimento do paciente (OPTION, *Observing Patient Involvement*) na tomada de decisões, para avaliar suas perspectivas sobre a TDC. Os escores médios da escala OPTION foram comparados usando testes *t* para amostras pareadas entre dentistas e pacientes, e as comparações intergrupos foram feitas usando testes *t* para amostras pareadas e coeficientes de correlação de Pearson.

**Resultados:**

Os escores OPTION foram semelhantes entre pacientes/pais e dentistas. No entanto, foram encontradas diferenças estatisticamente significativas nas questões sobre “diferentes fontes de informação”, “diferentes opções (incluindo a possibilidade de não fazer nada)” e “preocupações relativas à gestão do problema de saúde”, com os pacientes apresentando escores OPTION gerais mais baixos. Os pacientes deram escores OPTION mais baixos na TDC para o tratamento ortodôntico convencional, e escores mais altos para o tratamento ortopédico e a cirurgia ortognática, em comparação com os dentistas.

**Conclusões:**

O presente estudo revelou um consenso geral nos escores totais médios da escala OPTION entre pacientes e dentistas. Porém, quando estratificados, os pacientes apresentaram maiores escores de TDC para os casos ortopédicos e ortognáticos, e menores escores para o tratamento ortodôntico convencional.

## INTRODUCTION

Patient involvement in treatment decisions has received more attention in recent years.^1^ The likelihood for patient involvement and opinion exchange has increased due to the availability of informed consent, patient autonomy and shared approach.^2^ Now, the modern clinical practice is centered on patient satisfaction and convenience.^3^ This denotes the importance of incorporating clinician’s experience and patients values in the process of decision making, while taking into account the concept of evidence-based dentistry.^4^ There are various decision aid tools available that are helpful for delivering relevant information to the patient.^5^ However, they should be flexible enough to be used with patients who have various information requirements and want to be involved in decision-making process. Four widely used models are particularly prevalent in medical decision making, namely, paternalistic decision making, interpretive decision making, informed decision making and shared decision making (SDM).^6^

The SDM model has emerged as the primary objective of clinical practice, which is based on giving the patient all the knowledge and data that are accessible for a specific health issue and then assessing the efficacy of each treatment plan based on the patient’s preferences.^7^ SDM offers several key advantages. Firstly, it leads to more informed decisions by considering personalized treatment plans that aligns with patients’ goals and priorities. Secondly, it results in better patient outcomes, as it considers patients’ preferences and values. Thirdly, it enhances quality of life and respects patients’ chosen level of involvement, ultimately promoting to better adherence to treatment plans.^8^

There have been certain criteria laid down by Charles et al^6^ regarding SDM. According to him, the first attribute in SDM involves collaboration between the clinicians and patients. Family input could be crucial when choosing a course of treatment. Both parties should work together to reach an understanding over the preferred course of action. The clinician must describe the treatment options available and any potential side effects to the patient. Finally, the patient and the practitioner jointly should consider and assess the available therapeutic alternatives before reaching a conclusion on treatment implementation.^6^

Although SDM is a well-established approach in clinical medicine, there are only a handful of studies that have examined SDM in dentistry.^7^ Dentists require their patients to sign informed consent forms in addition to verbal consent as part of their legal and ethical responsibilities. As a result, it appears that SDM is used at the dental office. However, as it stands today, there are only a few investigations on SDM in dentistry, and just a few dental specializations have been the subject of this type of study.^9^ ^,^ ^10^ ^,^ ^13^ ^,^ ^14^

Several decision-making aids and support systems tailored to various dental specialties have been developed to contribute to the implementation of SDM in the dental field. These include patient decision-making aids (PDAs), that assist patients in reaching personal decisions regarding their health care options.^11^ As there are multiple treatment options available in the field of orthodontics, there is a greater need for SDM and PDAs. For this, one study analyzed Fixed Appliance Decision Aid (FADA), a new decision-making tool that not only included patients, but also their parents, and the results showed a high reliability of this tool.^12^

Another study examined the extent of SDM in orthodontics from the viewpoint of patients, clinicians and independent observers, using a 12-item dyadic questionnaire, observing patient involvement in decision making (OPTION).^13^ The results demonstrated that clinicians and patients perceived prominent levels of SDM, as compared to individual observers: the average OPTION scores for SDM were 90.4 ± 9.1%, 76.2 ± 8.95% and 42.6 ± 17.4%, respectively. However, the most significant factor was the patient’s perspective of SDM, because their care is impacted by their involvement.

Treatment recommendations mainly depends on patient’s age and dentofacial deformity. Conventional fixed appliance treatment suits mild to moderate skeletal discrepancies limited to dentition. Dentofacial orthopaedics suits growing patients with varied skeletal and dental issues, while severe skeletal problems in adults typically call for orthognathic surgery. Thus, the aim of the current study was to compare the patient’s perspective of SDM with that of clinicians, as assessed by the dyadic observing patient involvement (OPTION)^14^ scale, in patients needing dentofacial orthopaedics, orthognathic surgery or conventional non-surgical fixed appliance treatment.

## MATERIAL AND METHODS

A cross-sectional study was conducted at the Department of Orthodontics, Bakhtawar Amin Dental College and Hospital, Multan, Pakistan. The sample size was calculated for three groups using the findings of Keshtger et al^13^ who reported the mean dyadic OPTION score for patients and clinicians as 43.4+9.1 and 36.5+8.9, respectively. The alpha was set as 0.05 and power as 80% to calculate the sample size, which showed that was required a minimum sample size of 27 subjects in each group. To further increase the power of the study and to allow application of the central limit theorem, sample size was increased to 30 subjects in each group. Thus, the total sample for our study was 90 subjects. Ethical approval from the institutional ethical review board (ERC No: 1479-23) was obtained prior to data collection.

Inclusion criteria involved patients aged 10 to 45 years seeking orthodontic care in categories of orthopaedic appliances, orthodontic treatment with fixed mechanotherapy, and orthodontic treatment with orthognathic surgery. Growing patients with skeletal deformity and Angle Class II or III molar relationships were placed in the dentofacial orthopaedic group for treatment with growth modification appliances. The fixed mechanotherapy group included patients with ANB angle between 0° and 7°, with deformity primarily limited to dentition or with mild to moderate skeletal involvement. Adults with severe dentofacial deformities, having an ANB angle <-1° or >9°, and Angle Class II or III molar relationships were assigned to orthognathic surgery group. Patients with intellectual disability, language barriers, or acute mental health issues were excluded. Clinicians included specialist orthodontists and orthodontic residents who consented to participate.

The participants were selected using non-probability consecutive sampling techniques, where the data collection continued until required sample size was achieved in each treatment category. Informed consent was obtained from both the clinician and the parent/patient willing participate on the study. At the first appointment, the relevant history and pretreatment records were obtained. At the second appointment, a comprehensive treatment plan was presented to the patients. In case of patients younger than 18 years of age, the treatment plan was presented in the presence of a parent. After the consultation, the OPTION form (Table 1) in both Urdu and English was shared with the clinician and parent/patient, and their feedback was recorded.

**Table 1:** Total score of OPTION scale.

		Total score of OPTION scale Mean + SD	p
Patients gender^†^	Male (n=55)	39.328 ± 5.65	0.642
	Female (n=34)	38.936 ± 5.91	
Clinicians gender^†^	Male (n=55)	39.632 ± 4.87	0.104
	Female (n=35)	39.56 ± 2.90	
Patients’ socioeconomic status^‡^	Low income (n=28)	38.2 ± 5.52	0.395
	Middle income (n=46)	39.616 ± 5.69	
	High income (n=16)	39.68 ± 6.20	
Patients education status^‡^	Primary (n=16)	36.248 ± 5.46	0.038*
	Secondary (n=48)	39.864 ± 5.70	
	Graduation (n=21)	39.563 ± 5.52	
	Specialization (n=4)	40.8 ± 2.94	
Clinicians level^†^	Residents (n=46)	39.144 ± 6.36	0.105
	Consultants (n=44)	39.016 ± 6.41	

n = 90, SD = standard deviation. ^†^Independent sample t-test. ^‡^One-way ANOVA test. *p < 0.05.

Data was analyzed in SPSS for Windows (version 25.0, SPSS Inc. Chicago). The mean age of the participants and their gender distribution, socioeconomic status and educational background in each treatment category were calculated. The mean OPTION scale scores were compared between clinicians and patients/parents using paired sample *t*-test. The results were stratified according to the three treatment groups i.e. orthopaedic, orthodontic and orthognathic treatment. The intergroup comparisons among the three treatment groups were made using paired sample *t*-test along with their Pearson correlation coefficient. A*p*-value less than 0.05 was taken as statistically significant.

## RESULTS

A total of 90 patients participated in the study, out of which there were 56 females (62.2%) and 34 males (37.8%). The mean age of patients was 17.62 ± 6.21 years. The patient or their guardians (in case of a minor) were categorized according to their education status. Of the total sample, 16 (17.8%) were in the primary group, 48 (53.3%) in the secondary group, 22 (24.4%) in graduation and 4 (4.4%) in the specialization group. Characterization was also done regarding family’s monthly income in Pakistani rupees, into three categories: low <50,000 (31.1%), middle 50,000-150,000 (52.2%) and high 150,000 (15.6%).

The clinicians participating in the study included 35 females (38.9%) and 55 males (61.1%). The clinician category was divided into two groups, i.e.: residents 46 (51.1%) and consultants 44 (48.9%). 

Patients mean total OPTION scores were comparable for the consultations performed by male and female clinicians (*p*=0.642) or residents or specialists (*p*=0.852).

The gender of the clinician or patient and the socioeconomic status of the patient were not associated with the total score of OPTION scale (Table 1). However, there was a statistically significant difference in the mean total score for OPTION scale of patients among different groups, according to their education (*p*=0.030).

The mean score of the OPTION scale was 36.97+5.72 (70%) for patients and 36.97+4.15 (71%) for clinicians, with no statistically significant difference between them (*p* = 0.426). The overall comparison between mean scores of patients/parents with that of clinicians was found to be similar (Table 2). Statistical difference was found between the mean scores of patients and clinicians regarding Q3, related to provision of different sources of information to patients (*p*<0.05), with Pearson correlation coefficient r = 0.227. Statistical difference was also found in Q4, related to provision of different options to patient; and Q7, related to managing concerns and worries of patients, with a *p*<0.005 and r equal to 0.226 and 0.284, respectively. 

**Table 2:** Comparison between the mean score of OPTION scale between patients/parents and clinicians regarding SDM.

QUESTION	Score of OPTION scale Mean ± SD		p	r
	Patients (n=90)	Clinicians (n=90)		
1. A health problem related to my teeth/face was identified, where it was made clear that a decision was needed	3.40±0.73	3.45±0.62	0.614	-0.054
2. More than one way to manage health problem was described	3.02±0.88	3.02±0.91	0.365	0.097
3. Different sources of information (e.g leaflets, websites, contact with other people) to help make the decisions were offered	2.42±1.20	2.34±1.18	0.031*	0.227
4. Different options (including the possibility of doing nothing) were discussed	2.73±1.07	2.61±1.04	0.032*	0.226
5. The advantages, disadvantages and possible outcomes of options were discussed	3.19±0.76	3.21±0.62	0.429	-0.084
6. Ideas or expectations about managing the health problem were discussed	2.92±0.96	3.09±0.69	0.492	-0.073
7. Concerns or worries about managing the health problem were discussed	2.91±0.93	3.08±0.70	0.007*	0.284
8. It was made sure that information had been understood	3.42±0.56	3.49±0.50	0.875	0.017
9. There were opportunities to ask questions	3.41±0.49	3.46±0.63	0.962	0.005
10. The preference to take part in the decision (or not) was respected	3.47±0.52	3.46±0.60	0.166	-0.147
11. During the consultation, a decision was made or there was an agreement to postpone making the decision	2.93±1.00	3.17±0.81	0.897	0.014
12. The possibility of coming back to the decision was discussed	3.13±0.73	3.14±0.66	0.785	0.029
Total score (48)	36.97±5.72	36.97±4.15	0.426	0.446

n = 90. Paired sample t-test. Pearson correlation.

**Figure 1:**
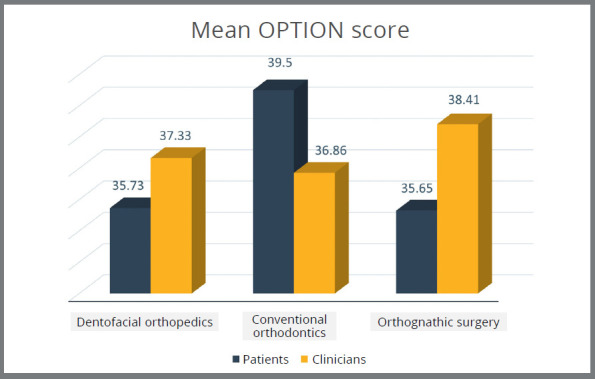
Comparison of mean OPTION scores between patients and clinicians for different treatment categories.

Comparison between the mean scores of OPTION scale between patients/parents and clinicians regarding SDM among various treatment modalities was also done (Fig 1). A statistically significant difference in the dentofacial orthopaedic treatment modality was identified in Q3 and Q7, with*p*<0.005 and r value of 0.130 and 0.412, respectively (Table 3). 

**Table 3:** Comparison between the mean score of OPTION scale between patients/parents and clinicians regarding SDM in patients needing Dentofacial Orthopedics

QUESTION	Score of OPTION scale Mean±SD		p	r
	Patients (n=30)	Clinicians (n=30)		
1. A health problem related to my teeth/face was identified, where it was made clear that a decision was needed	3.40±1.03	3.53±0.50	0.581	0.105
2. More than one way to manage health problem was described	2.87±0.93	3.03±0.89	0.367	0.390
3. Different sources of information (e.g leaflets, websites, contact with other people) to help make the decisions were offered	2.33±1.15	2.30±1.14	0.033*	0.130
4. Different options (including the possibility of doing nothing) were discussed	3.47±1.10	2.37±1.12	0.678	0.367
5. The advantages, disadvantages and possible outcomes of options were discussed	3.00±1.05	3.17±0.79	1.000	0.000
6. Ideas or expectations about managing the health problem were discussed	2.80±1.15	3.07±0.82	0.623	0.094
7. Concerns or worries about managing the health problem were discussed	2.80±1.06	3.00±0.78	0.024*	0.412
8. It was made sure that information had been understood	3.47±0.50	3.57±0.50	0.962	0.009
9. There were opportunities to ask questions	3.43±0.50	3.47±0.81	0.977	0.006
10. The preference to take part in the decision (or not) was respected	3.47±0.50	3.43±0.67	0.574	0.107
11. During the consultation, a decision was made or there was an agreement to postpone making the decision	2.77±1.04	3.27±0.78	0.167	0.259
12. The possibility of coming back to the decision was discussed	2.93±0.86	3.13±0.77	0.542	0.116
Total score (48)	35.73±5.98	37.33±4.17	0.450	0.143

n=30. Paired sample *t*-test. Pearson correlation.

Regarding conventional orthodontics, a statistical difference was noted in Q5, which was related to provision of different advantages, disadvantages and possible options to the patients, with p<0.005 and negative correlation of -0.445 (Table 4). In relation to orthognathic surgery, Q3 and Q4 showed a statistically significant difference, with p<0.005. Their r value was found to be 0.411 and 0.769, respectively (Table 5).

[Table t4]

**Table 4: t4:** Comparison between the mean score of OPTION scale between patients/parents and clinicians regarding SDM in patients needing Conventional Orthodontics.

QUESTION	Score of OPTION scale Mean±SD		p	r
	Patients (n=30)	Clinicians (n=30)		
1. A health problem related to my teeth/face was identified, where it was made clear that a decision was needed	3.57±0.50	3.30±0.79	0.154	0.267
2. More than one way to manage health problem was described	3.27±0.78	2.90±0.99	0.515	0.058
3. Different sources of information (e.g leaflets, websites, contact with other people) to help make the decisions were offered	2.50±1.16	2.23±1.27	0.762	0.073
4. Different options (including the possibility of doing nothing) were discussed	3.07±0.74	2.73±0.98	0.702	0.124
5. The advantages, disadvantages and possible outcomes of options were discussed	3.37±0.55	3.07±0.52	0.014*	0.445
6. Ideas or expectations about managing the health problem were discussed	3.23±0.62	2.97±0.71	0.260	0.212
7. Concerns or worries about managing the health problem were discussed	3.00±0.91	3.07±0.45	0.178	0.253
8. It was made sure that information had been understood	3.57±0.67	3.47±0.50	0.972	0.007
9. There were opportunities to ask questions	3.60±0.49	3.50±0.50	1.000	0.000
10. The preference to take part in the decision (or not) was respected	3.67±0.47	3.37±0.61	0.411	0.156
11. During the consultation, a decision was made or there was an agreement to postpone making the decision	3.30±0.70	3.17±0.69	0.353	0.176
12. The possibility of coming back to the decision was discussed	3.37±0.49	3.10±0.66	0.538	0.117
Total score (48)	39.50±4.84	36.86±3.88	0.893	0.026

n=30. Paired sample *t*-test. Pearson correlation.

**Table 5:** Comparison between the mean score of OPTION scale between patients/parents and clinicians regarding SDM in patients needing Orthognathic Surgery.

QUESTION	Score of OPTION scale Mean±SD		p	r
	Patients (n=30)	Clinicians (n=30)		
1. A health problem related to my teeth/face was identified, where it was made clear that a decision was needed	3.24±0.51	3.52±0.50	0.788	0.052
2. More than one way to manage health problem was described	2.93±0.90	3.13±0.86	0.769	0.376
3. Different sources of information (e.g leaflets, websites, contact with other people) to help make the decisions were offered	2.43±1.33	2.50±1.13	0.041*	0.411
4. Different options (including the possibility of doing nothing) were discussed	2.67±1.26	2.73±1.01	0.024*	0.769
5. The advantages, disadvantages and possible outcomes of options were discussed	3.20±0.55	3.40±0.49	0.692	0.075
6. Ideas or expectations about managing the health problem were discussed	2.73±0.98	3.23±0.50	0.289	0.200
7. Concerns or worries about managing the health problem were discussed	2.93±0.82	3.17±0.83	0.379	0.167
8. It was made sure that information had been understood	3.23±0.43	3.43±0.50	0.978	0.005
9. There were opportunities to ask questions	3.20±0.40	3.40±0.56	0.752	0.060
10. The preference to take part in the decision (or not) was respected	3.27±0.52	3.57±0.50	0.713	0.070
11. During the consultation, a decision was made or there was an agreement to postpone making the decision	2.73±1.14	3.07±0.94	0.102	0.305
12. The possibility of coming back to the decision was discussed	3.10±0.75	3.20±0.55	0.863	0.033
Total score (48)	35.65±5.59	38.41±4.39	0.207	0.241

n=30. Paired sample t-test. Pearson correlation.

## DISCUSSION

The current study is the first of its kind to investigate the level of SDM among different treatment modalities encountered in the field of Orthodontics, in this particular population. The general comparison between patient and clinician’s mean OPTION scores revealed an overall consensus between the patients and clinicians regarding the practice of SDM (Table 2). After stratification, the results showed a slightly different scenario, showing an overall greater patient OPTION scores for orthopaedic (Table 2) and orthognathic patients (Table 4), and smaller overall patient OPTION scores for conventional orthodontic treatment (Table 3).

The relationship of the overall patient OPTION score with patients’ and clinicians’ gender, patients’ socioeconomic status and education level and clinician’s level was also explored in this study (Table 1). Amongst all these factors, only patients’ education level was found to significantly affect the overall patient OPTION score, that highlight the importance of modifying patient counseling technique for patients with varying levels of knowledge.

SDM has been proposed as the “appropriate ideal for patient-professional relationships that a sound doctrine of informed consent should support”, by the US Presidential Commission on Medical Decision Making Ethics.^16^ As it involves an interpersonal and interdependent process between the both parties, the concept of SDM is crucial for the overall satisfaction with treatment in general.^17^ The use of OPTION scale has proved to be a valid and reliable measure for SDM in clinical settings.^14^ Only a few studies has been reported on SDM using the OPTION scale, and none in particular for the current population.^9^ ^,^ ^10^ The results showing a higher standard of SDM in this particular population were encouraging. A similar study conducted by Keshtgar et al^13^ also reported a high SDM level in their population, and the differences noted were largely due to the further stratification done in the current study, based on specific treatment modalities.

The total patient and clinician OPTION scores were reported to be very similar upon comparison in non-stratified results, which shows a general confidence in shared decision making by the patients. Statistically significant differences were found regarding the questions about “different sources of information” (Q.3), “advantage, disadvantage and possible outcomes” (Q.4) and “concerns regarding management” (Q.7), with the patients giving overall smaller OPTION scores. This may be because the consultations had to be done in a limited time span, due to the setting being a general outpatient department, which led to a smaller OPTION score by the patients. Time constraints are a frequently reported barrier to clinical changes, including SDM.^18^

Interestingly, the clinicians gave a smaller OPTION score to Q.7 than the patients, which may indicate a general lack of patient enthusiasm and involvement felt by the clinicians during consultations, probably due to low internal motivation in some orthodontic patients. Patients may be unwilling to participate in decision-making, due to a lack of self-efficacy rather than a genuine disinterest.^19^

Surprisingly, the patients reported lesser OPTION scores for SDM regarding conventional orthodontic treatment than the clinicians, which indicates an overestimation of the quality of SDM provided by the clinicians when dealing with conventional orthodontic treatment cases. A cross-sectional study conducted on adult orthodontic patients found that they perceive a more passive role in their current treatment decisions, rather than preferring SDM.^20^

Current findings establish the need to emphasize the SDM process when dealing with routine or less complex orthodontic cases, to match them to more demanding clinical scenarios and to avoid any negligence or bias from the clinician’s perspective.

Furthermore, these findings bring to light the general lack of awareness and information regarding orthodontic treatment among the general population, which might be leading to lower OPTION scores among the patients. An increasing number of countries are now choosing to focus their policy decisions on the patient, in view of the current shift in patient-centered care and the possible systemic benefits revealed by recent research on SDM.^21^

The patient OPTION scores for SDM regarding orthopaedic treatment and orthognathic surgery were reported to be higher than those of the clinicians, which indicates an overall satisfaction with the level of SDM being provided in the current setting. These findings also point towards a greater concern and involvement of the patients regarding orthopaedic and orthognathic treatment modalities, which need to be promptly addressed by the clinicians.

The current study was limited by the lack of a bigger sample size of the population, as well as by the absence of an additional third party for the OPTION scoring, which would increase the overall reliability and validity of the results.

## CONCLUSIONS

A broad consensus between patients and clinicians was found in the current study, which examined the dynamics of SDM across different orthodontic treatments. However, upon stratification, a more detailed picture emerged. Notably, patients exhibited higher SDM scores in cases involving orthopaedic and orthognathic treatments, suggesting a greater collaborative decision-making process in these complex interventions. In contrast, lower SDM scores were observed in the context of conventional orthodontic treatment, hinting at potential areas for improvement in patient-clinician communication and involvement in more routine procedures. 

## References

[1] Coulter A (2017). Shared decision making everyone wants it, so why isn't it happening?. World Psychiatry.

[2] AlSarhan MA, Alaqeely RS, AlJasser R, Otaibi DH, AlOraini S, Alshiddi IF (2021). Evaluation of complacency about dental implants with shared decision making and satisfaction scores a cross-sectional study. Saudi Dent J.

[3] Coulter A, Edwards A, Elwyn G, Thomson R (2011). Implementing shared decision making in the UK. Z Evid Fortbild Qual Gesundhwes.

[4] Nagendrababu V, Vinothkumar TS, El-Karim I, Rossi-Fedele G, Dogramaci EJ, Dummer PMH (2023). Dental patient-reported outcomes in endodontics - a narrative review. J Evid Based Dent Pract.

[5] Scalia P, Durand MA, Berkowitz JL, Ramesh NP, Faber MJ, Kremer JAM (2019). The impact and utility of encounter patient decision aids systematic review, meta-analysis and narrative synthesis. Patient Educ Couns.

[6] Charles C, Gafni A, Whelan T (1997). Shared decision-making in the medical encounter what does it mean?(or it takes at least two to tango). Social science & medicine.

[7] Park SG, Lee S, Kim MK, Kim HG (2012). Shared decision support system on dental restoration. Expert Syst Appl.

[8] Joseph-Williams N, Lloyd A, Edwards A, Stobbart L, Tomson D, Macphail S (2017). Implementing shared decision making in the NHS lessons from the MAGIC programme. BMJ.

[9] Asa'ad F (2019). Shared decision-making (SDM) in dentistry a concise narrative review. J Eval Clin Pract.

[10] Huang YK, Chen YT, Chang YC (2022). The implementation of shared decision-making in clinical dentistry opportunity and change. J Formos Med Assoc.

[11] Parker K, Cunningham SJ, Petrie A, Ryan FS (2017). Randomized controlled trial of a patient decision-making aid for orthodontics. Am J Orthod Dentofacial Orthop.

[12] Marshman Z, Eddaiki A, Bekker HL, Benson PE (2016). Development and evaluation of a patient decision aid for young people and parents considering fixed orthodontic appliances. J Orthod.

[13] Keshtgar A, Cunningham SJ, Jones E, Ryan FS (2021). Patient, clinician and independent observer perspectives of shared decision making in adult orthodontics. J Orthod.

[14] Joosten EA, DeFuentes-Merillas L, de Weert GH, Sensky T, van der Staak CP, de Jong CA (2008). Systematic review of the effects of shared decision-making on patient satisfaction, treatment adherence and health status. Psychother Psychosom.

[15] Elwyn G, Edwards A, Wensing M, Hood K, Atwell C, Grol R (2003). Shared decision making developing the OPTION scale for measuring patient involvement. Qual Saf Health Care.

[16] United States (1978). President's Commission for the Study of Ethical Problems in Medicine and Biomedical and Behavioral Research.

[17] Légaré F, Witteman HO (2013). Shared decision making examining key elements and barriers to adoption into routine clinical practice. Health Aff.

[18] Légaré F, Ratté S, Gravel K, Graham ID (2008). Barriers and facilitators to implementing shared decision-making in clinical practice update of a systematic review of health professionals' perceptions. Patient Educ Couns.

[19] Légaré F, St-Jacques S, Gagnon S, Njoya M, Brisson M, Frémont P (2011). Prenatal screening for Down syndrome a survey of willingness in women and family physicians to engage in shared decision-making. Prenat Diagn.

[20] Motamedi-Azari F, Ryan FS, Jones E, Cunningham SJ (2020). A cross-sectional study investigating patients' preferences regarding shared decision-making in adult orthodontic patients. Br Dent J.

[21] Härter M, van der Weijden T, Elwyn G (2011). Policy and practice developments in the implementation of shared decision making an international perspective. Z Evid Fortbild Qual Gesundhwes.

